# Change in microbiota profile after vaginal estriol cream in postmenopausal women with stress incontinence

**DOI:** 10.3389/fmicb.2024.1302819

**Published:** 2024-03-05

**Authors:** Kate H. Moore, Samantha Ognenovska, Xin-Yi Chua, Zhuoran Chen, Chloe Hicks, Fatima El-Assaad, Nevine te West, Emad El-Omar

**Affiliations:** ^1^Department of Urogynaecology, St George Hospital, University of New South Wales, Sydney, NSW, Australia; ^2^University of New South Wales Microbiome Research Centre, St George and Sutherland Clinical Campuses, School of Clinical Medicine, Faculty of Medicine and Health, University of New South Wales, Sydney, NSW, Australia

**Keywords:** vaginal estrogen, vaginal microbiota, lactobacilli, menopause, stress incontinence

## Abstract

**Introduction:**

Vaginal estrogen is a treatment for genitourinary symptoms of menopause (GSM), which comprises vaginal atrophy and urinary dysfunction, including incontinence. Previous studies show that estrogen therapy promotes lactobacilli abundance and is associated with reduced GSM symptoms, including reduction of stress incontinence. However, detailed longitudinal studies that characterize how the microbiome changes in response to estrogen are scarce. We aimed to compare the vaginal microbiota of postmenopausal women, before and 12 weeks after vaginal estrogen cream.

**Methods:**

A total of 44 paired samples from 22 postmenopausal women with vaginal atrophy and stress incontinence were collected pre-vaginal estrogens and were compared to 12 weeks post-vaginal estrogen. Microbiota was characterized by 16S rRNA amplicon sequencing and biodiversity was investigated by comparing the alpha- and beta-diversity and potential markers were identified using differential abundance analysis.

**Results:**

Vaginal estrogen treatment was associated with a reduction in vaginal pH and corresponded with a significant reduction in alpha diversity of the microbiota. Healthy vaginal community state type was associated with lower mean pH 4.89 (SD = 0.6), in contrast to dysbiotic state which had a higher mean pH 6.4 (SD = 0.74). Women with lactobacilli dominant community pre-treatment, showed stable microbiota and minimal change in their pH. Women with lactobacilli deficient microbiome pre-treatment improved markedly (*p* = 0.004) with decrease in pH −1.31 and change to heathier community state types.

**Conclusion:**

In postmenopausal women with stress incontinence, vaginal estrogen promotes *Lactobacillus* and *Bifidobacterium* growth and lowers vaginal pH. Maximum response is seen in those with a dysbiotic vaginal microbiota pre-treatment.

## 1 Introduction

As the median lifespan of women continues to rise, Genitourinary Syndrome of the Menopause (GSM) is becoming an increasing problem, affecting 45–77% of post-menopausal women (Santoro and Komi, 2009; Minkin et al., 2015). The syndrome comprises post-menopausal symptoms of vaginal atrophy (dryness/soreness/dyspareunia), along with intermittent vaginal discharge and urinary dysfunction, which may include both stress and urge urinary incontinence. Although systemic Hormone Replacement Therapy (HRT) often benefits vaginal atrophy, systemic HRT is of no benefit for urinary symptoms and may in fact precipitate or worsen incontinence (Manson et al., 2013). Therefore, women with post-menopausal incontinence are commonly treated with topical vaginal estrogen cream (estriol) or vaginal tablets (estradiol; Cody et al., 2012).

In recent years, interest in the vaginal microbiome has increased, particularly regarding changes observed in bacterial biodiversity after menopause. Initial studies in the early 2000's explored the microbiome using traditional culture methods, via identification of *Lactobacillus* in the vagina (Yoshimura and Okamura, 2001; Heinemann and Reid, 2005). Systemic HRT for 2 weeks was associated with increased *Lactobacillus* detection in the vagina. Subsequently, Shen et al. investigated the impact of systemic HRT via “low dose estrogen therapy” on the vaginal microbiota of post-menopausal women, and using 16S rRNA amplicon sequencing, showed similar findings (Shen et al., 2016). Due to more recent understanding of the difference between HRT and vaginal estrogen, Gliniewicz et al. studied 15 post-menopausal women having no therapy and 15 women having unspecified therapy (either oral tablets, transdermal patch or vaginal therapy not stated) for the association between GSM and estrogen use (Gliniewicz et al., 2019). But these authors collected only one vaginal swab for their 16S rRNA studies after estrogen use and thus no baseline microbiome data was available to detect any post treatment changes. They did show increased predominance of *Lactobacillus* within the vaginal microbiota in the group who received estrogen therapy, and a decrease in vaginal pH (Shen et al., 2016; Gliniewicz et al., 2019).

Thus, the aim of this study was to conduct 16S rRNA amplicon sequencing in women with GSM and stress incontinence both before and after vaginal estrogen topical therapy.

## 2 Materials and methods

### 2.1 Patients and sample collection

Post-menopausal women >51 yrs old who had a main complaint of stress urinary incontinence (SUI; i.e., leakage with coughing, sneezing, exercise, or lifting), who also had symptoms and visual evidence of vaginal atrophy were prospectively recruited after informed consent in a single center. Exclusion criteria included women undergoing other treatments for their SUI, or currently using other forms of topical estrogen or hormone replacement (or having used this within the 12 months prior), a history of breast cancer, or women who could not give consent in English without an interpreter.

Study treatment involved digital application of 0.5 mg of 1 mg/g estriol (E3) cream (Ovestin^®^) around the opening of the urethra and within the vaginal opening for 12 weeks; daily for the first 3 weeks, then reduced to three times per week thereafter. Adherence to the estriol cream instructions was checked with a compliance diary. Estriol crème (Ovestin) applied to the suburethral vagina was chosen, rather than estradiol tablets (Vagifem) placed high in the vagina, because the former has less likelihood of promoting endometrial hyperplasia, and application to the suburethral vagina is more likely to enhance local mucosal proliferation and co-adaptation of the mucosa, to ameliorate stress incontinence (Te West et al., 2023).

A standard mid vaginal swab (ESwabTM, Copan Diagnostics, USA) containing 1 mL of liquid Amies media was collected both pre- and post-treatment. Swabs were stored at −80°C within 30 min of collection. Additionally, vaginal pH was recorded at baseline and follow up. A litmus strip was used with a range of 4.0–7.0 and intervals of 0.2–0.3 (mColorpHast TM strips). The strip was inserted inside the vagina for 20 s and then dried for 60 s, before comparing the color on the strip to a standardized chart from the manufacturer and the pH was noted. A reduction in vaginal pH suggests that vaginal atrophy has been reduced and that maturation of the vaginal epithelium has improved.

Incontinence outcomes including erect cough stress test, International Consultation on Incontinence Questionnaire (ICIQ) were measured at both time points. The present study of microbiome characterization was a subset of a larger clinical trial regarding the benefit of vaginal estriol for stress incontinence in a larger cohort of post-menopausal women, which has previously been published (Te West et al., 2023). Ethics approval for the main study and the sub-study was obtained from the local hospital Research Ethics Committee (LNR/13/POWH/685). Written consent was obtained from all participants.

### 2.2 Microbiota DNA extraction and sequencing

Swab samples were thawed on ice and DNA extraction was performed as per Susic et al. using the QIAamp DNA Mini Kit (Qiagen, USA; Susic et al., 2020). One additional step was included (as per Gliniewicz et al., 2019); after enzymatic lysis and incubation, samples were mechanically lysed using 0.3 g of 0.1 mm zirconia beads and Tissuelyser II (Qiagen, USA) at 30 Hz for two 1-min intervals (samples on ice for 1 min in-between). Extracted DNA was quantified using a Qubit™ dsDNA BR Assay kit (Invitrogen, USA) and Qubit 2.0 Fluorometer (Life Technology, USA). DNA samples were subsequently sent to the UNSW Ramaciotti Center for Genomics, whereby the V3-V4 hypervariable region of the 16S rRNA gene were targeted for amplicon sequencing using the 341F-805R primer pair. Samples were sequenced on Illumina MiSeq, generating paired end 300 bp reads. The V3-V4 hypervariable region is a common 16S rRNA region used for surveying the vaginal microbiota with some studies showing that it can detect more vaginal microbial diversity than V1-V2 hypervariable regions (Graspeuntner et al., 2018; Van Der Pol et al., 2019; Hugerth et al., 2020).

### 2.3 Data processing

Sequencing data were processed following the QIIME2 pipeline (Bolyen et al., 2019) with the DADA2 (Callahan et al., 2016) plugin. More specifically, sequenced reads were denoised, de-replicated and filtered for chimeric reads using default parameter settings and the following specific parameter settings for trimming of reads: forward truncation = 290, reverse truncation = 220, forward-trim = 20, and reverse-trim = 8. The reads were then clustered to generate amplicon sequence variants (ASVs) as per the algorithm by DADA2. Representative reads for each ASV were checked for any host DNA by aligning against the human genome (GRCh38) using bowtie2 (Langmead and Salzberg, 2012) and removed when detected. After decontamination, each remaining representative ASV read were taxonomically assigned using a Naïve Bayes classifier, which was trained following the QIIME2 “Training feature classifiers with q2-feature-classifiers” tutorial by extracting the 16S rRNA V3-V4 hypervariable region from the Greengenes (DeSantis et al., 2006) database (release 13_5). A phylogenetic tree was then constructed following the QIIME2 pipeline. A taxonomy abundance table was generated for diversity analyses. Data was normalized by rarefaction to a sample depth of 10,000 reads per sample for alpha- and beta-diversity analysis. The full data set was investigated for exploratory analysis and differential abundance analysis.

### 2.4 Data analysis

Statistical analysis was performed in R v4.0.2 (RC, 2018) to compare alpha-diversity and beta-diversity between pre- and post-treatment. Alpha-diversity of the vaginal microbiota pre and post vaginal estrogen therapy was assessed using different alpha diversity metrics with R packages vegan v2.5–7 (Oksanen et al., 2022) and otuSummary v0.1.1 (Yang, 2020). Tests for group differences were carried out with Wilcoxon signed-rank test for paired samples. Correlations between alpha-diversity and vaginal pH was performed using repeated measures correlation R package, rmcorr v0.4.6 (Bakdash and Marusich, 2017). Comparisons of compositional differences pre vs. post estrogen were examined with beta diversity measured by Bray-Curtis dissimilarity, performed using the Permutational Multivariate Analysis of Variance (PERMANOVA; Anderson, 2017) method implemented in the adonis2 function in vegan. Detection of differentially abundant taxa was carried out using Linear discriminant analysis Effect Size (LEfSe; Segata et al., 2011). Plots were generated using the R packages ggplot2 (Wickham, 2009), ggpubr (Kassambara, 2020), and cowplot (Wilke, 2020).

#### 2.4.1 Subgroup analysis

Subgroup analysis was performed comparing microbiota change based on patients change in vaginal pH, with two subgroups identified. Patients whose pH was reduced in response to vaginal estrogen were labeled *responders*, and whose pH remained the same or increased were termed *non-responders*.

#### 2.4.2 Hierarchical clustering and community state types (CSTs)

Samples were hierarchically clustered using complete linking with the Bray-Curtis dissimilarity values as input. The dendrogram was visualized along with the relative abundance heatmap of the top 20 most dominant genera across all samples. This allowed assessments of the natural groupings of similar samples and how they correlate with vaginal pH and treatment timepoint. Additionally, samples were assigned to community state types (CSTs) based on the dominant taxa in each sample according to previous research (Ravel et al., 2011; De Seta et al., 2019; Mancabelli et al., 2021). The CST groups: CST I–III, V (dominant in *Lactobacillus* spp.); CST-Bifido (*Bifidobacterium*); CST IV-A (*Gardnerella* with relative abundance ≥50%); CST IV-B (*Gardnerella* with relative abundance <50%); CST Mix-A (consists of *Anaerococcus, Peptoniphilus, Prevotella*, and *Streptococcus*); CST Mix-B (*Atopobium* and *Megasphera*) and CST-NA (remaining samples that do not fit into one of the above states). Literature searches were subsequently performed to determine the microbial role of the dominant taxa identified within these patients using search terms: “name of bacterial taxa” AND “vagina” AND “role OR pathogen OR commensal.” Search results were reviewed, and the bacterial taxa grouped according to the results of multiple clinical trials as: possessing a beneficial, pathogenic, dual or un-determined role within the vagina.

## 3 Results

### 3.1 Study population and samples analyzed

**Figure 1:**
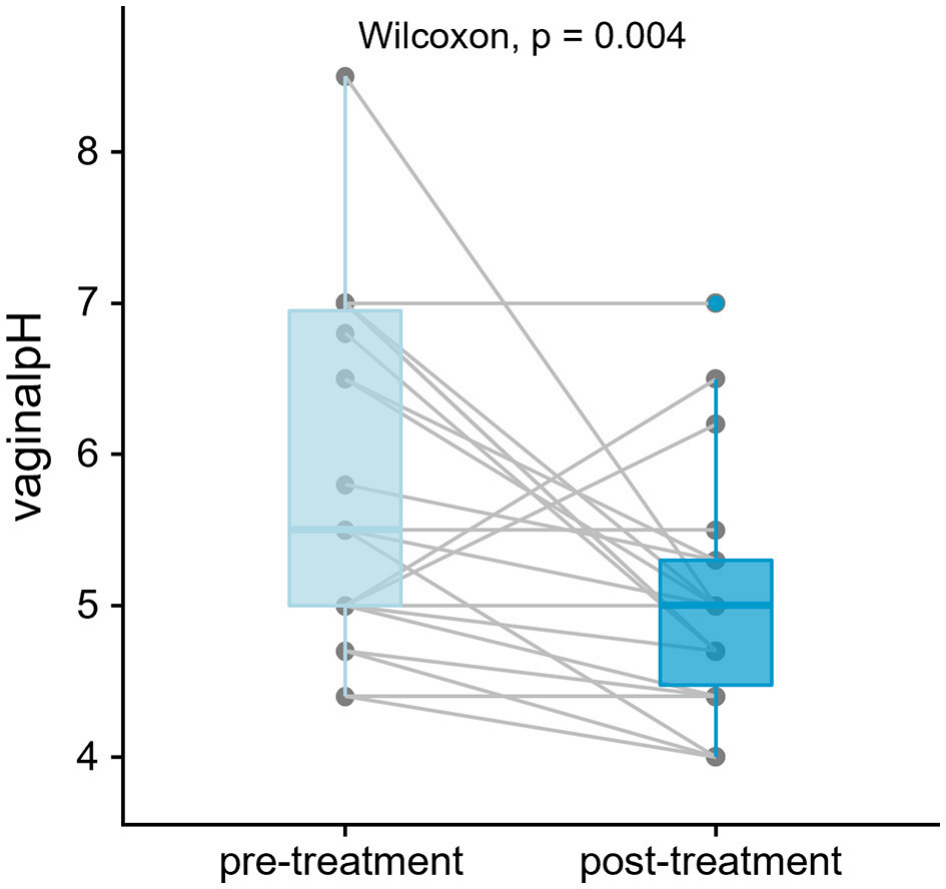
Vaginal pH has significantly decreased post-treatment (*p* < 0.05, *n* = 22 patients, 44 samples). One-tailed paired test was performed using Wilcoxon signed-rank. Lines join measurements taken from the same subject.

A total of 44 paired samples from 22 patients were analyzed for the study, obtained from post-menopausal women with stress incontinence and vaginal atrophy who had no prior vaginal estrogen and no recent systemic HRT. Patient demographics are shown in Table 1. After quality control, 36 paired samples remained. The vaginal pH showed a statistically significant reduction after estrogen cream use: vaginal pH fell from 5.5 to 5.0 (4.8–5.3) [Median (IQR), *p* = 0.004 Wilcoxon signed-rank test] (Figure 1). The objective outcomes for stress incontinence were analyzed, showing a trend toward reduction in leakage amount, but results were not statistically significant owing to the small sample size. However, the estriol benefit did achieve significance in the large “parent” cohort (Te West et al., 2023).

**Table 1:** Summary of cohort characteristics in this study.

**Patient characteristics**	***N***	**Median (IQR)**
Age	22	62.7 (58.1–65.1)
BMI	21	27.8 (25.2–31.3)
Ethnicity		n/a
Caucasian	20	
Asian	1	
Indian	1	
Vaginal pH at pre-treatment	22	5.5 (5.0–6.9)
Vaginal pH at post-treatment	22	5.0 (4.8–5.3)

Fifteen women showed decrease in their vaginal pH post-treatment and were labeled as responders; and remaining seven showed either no change or an increase in vaginal pH post-treatment and were labeled as non-responders.

### 3.2 Overview of vaginal microbiota

**Figure 2:**
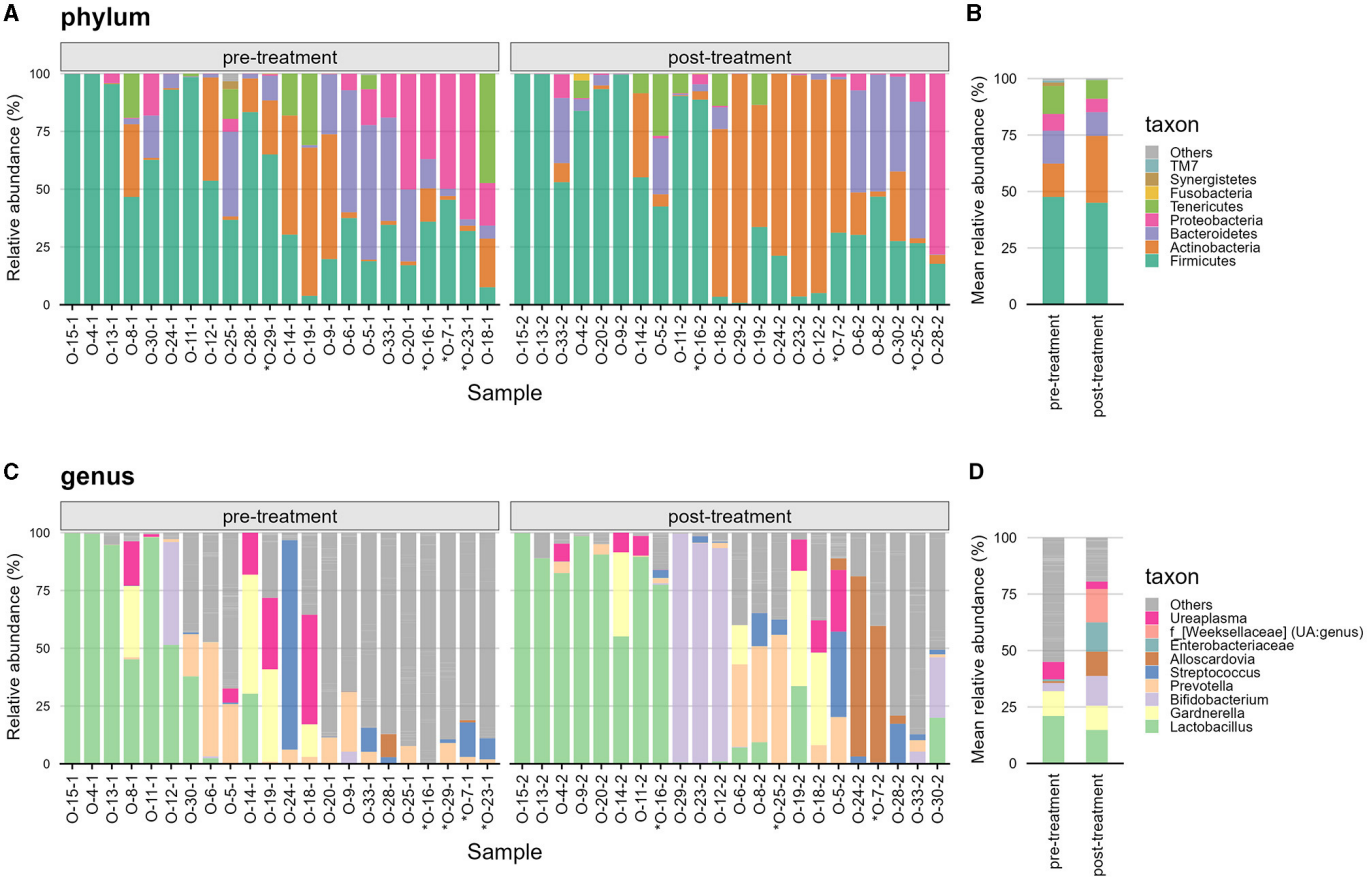
Overview of taxonomy landscape of the vaginal microbiota in this study. **(A)** Shows the top seven phyla colored and the remaining non-top seven phyla aggregated to “Others” in gray. Samples are separated by pre-treatment on the left and post-treatment on the right. **(B)** Shows the mean relative abundance at phylum rank comparing pre- vs. post-treatment. **(C, D)** Shows similar information at the Genera rank with only the top seven genera colored and the remaining non-top seven genera aggregated to “Others;” with samples in **(C)** and grouped by treatment in **(D)**. Samples marked with asterisks (^*^) had <10,000 reads and were removed by rarefaction normalization for alpha-diversity analyses but included for downstream exploratory data analysis.

A total of 4.1 million reads were sequenced of which after quality control, 2.2 million reads remained with an average of ca. Fifty-one thousand three hundred reads per sample. There was a total of 1,266 amplicon sequence variants (ASVs) detected which were annotated to 163 genera belonging to 18 phyla (Figure 2). The number of detected genera range from 5 to 76 with a mean of 34.3 (SD = 19.7) genera per sample.

### 3.3 Samples cluster with higher vaginal pH

**Figure 3:**
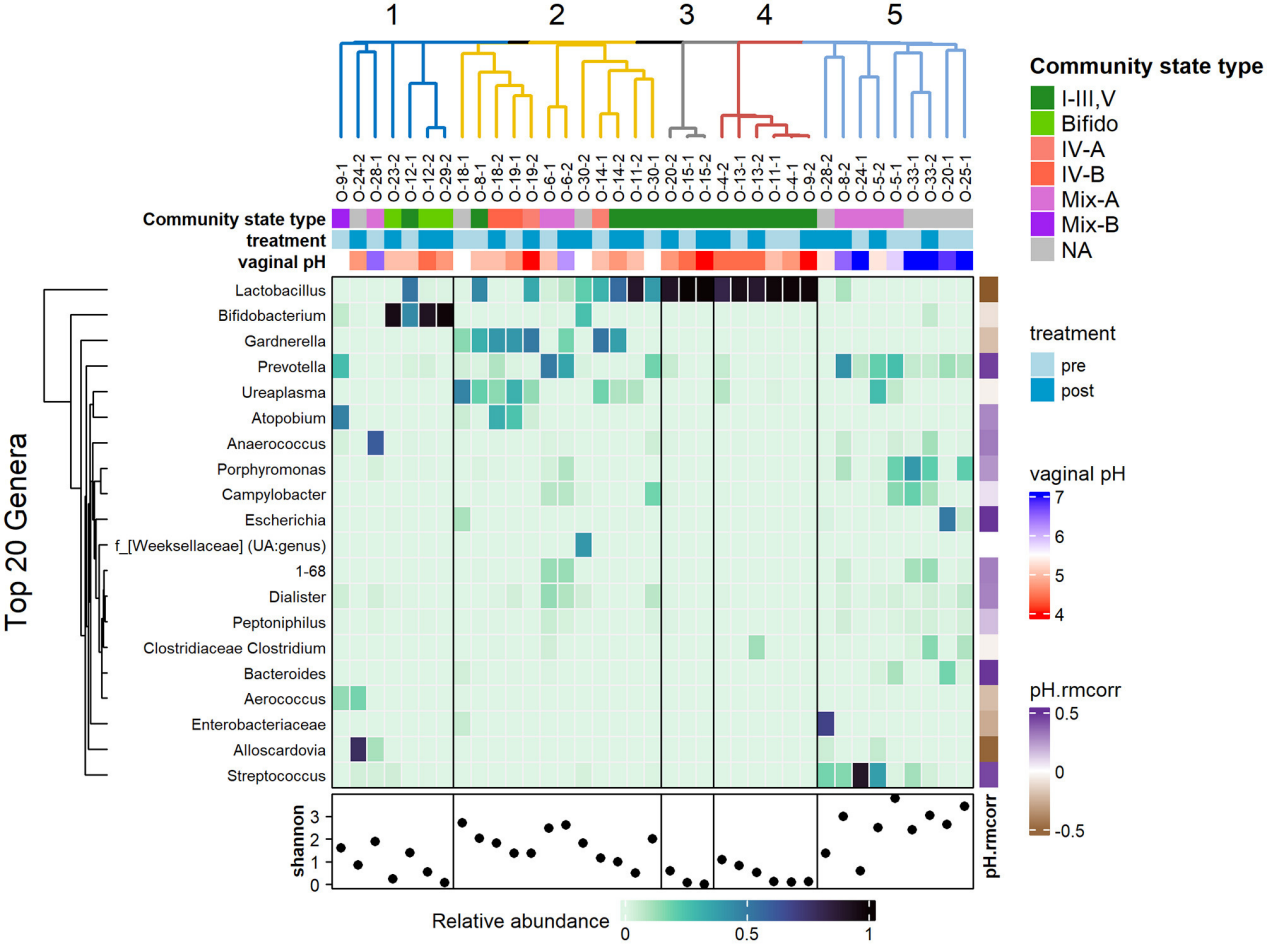
Heatmap showing the relative abundance of the top 20 genera, clustered using hierarchical clustering of Bray-Curtis dissimilarity. Top annotations: hierarchical clustering of samples; community state type of samples based on most dominant taxa; treatment timepoints and vaginal pH. Samples clusters are highly associated with vaginal pH rather than treatment timepoint. The bottom row shows the Shannon diversity for each sample with the fifth cluster showing higher diversity and higher vaginal pH. The vertical annotation on the right (ph.rmcorr) shows the repeated correlation coefficient of each genus with respect to the vaginal pH where the purple shades show positive correlation and brown shades show negative correlation.

Hierarchical clustering of samples based on compositional similarity returned five major clusters. There were no noticeable patterns of clusters with relation to the pre- or post-treatment timepoints. Instead, as shown in Figure 3, clusters were well-aligned with vaginal pH. Clusters 1–4 of generally healthy vaginal communities were associated with 28 samples with mean vaginal pH of 4.89 (SD = 0.6). In contrast, cluster 5 was associated with nine samples with mean vaginal pH of 6.4 (SD = 0.74).

Visually, in Figure 3, the clusters are organized by the most dominant taxa, with cluster 1 associated with high abundance of *Bifidobacterium*; cluster 2 is largely associated with a mix of *Gardnerella, Ureaplasma, Lactobacillus*, and *Atopobium*; clusters 3 and 4 are associated with very high abundance of *Lactobacillus*; and cluster 5 had a lack of previously mentioned taxa. Cluster 5 showed higher biodiversity, as indicated by the higher Shannon index values, and was populated by a mix of *Streptococcus, Prevotella, Porphyromas*, and *Campylobacter*. The clusters coincided with the supervised partitioning of samples into community state types (CSTs) based on the most dominant species. Clusters 3 and 4 are associated with CST I–III, V (dominant in lactobacilli); and cluster 2 and 5 had the most variable CST with mixtures of IV-A, IV-B, Mix-A, Mix-B, and CST-NA, representing the more diverse state types.

Correlation of the relative abundance of the top 20 genera against vaginal pH was measured using repeated measures correlation with the rmcorr R package. As expected, *Lactobacillus* showed negative correlation with vaginal pH (*r* = −0.57, *p* = 0.01, not significant after adjustment for multiple comparison of the genus). Negative correlation is also noted with *Bifodobacterium, Gardnerella, Enterobacteriaceae*, and *Alloscardia*, albeit the latter few are only present in a couple of samples, thus reviewing these taxa with a larger sample size would be ideal. *Streptococcus, Bacteroides, Escherichia*, and *Prevotella* (coefficients ranging from 0.44 to 0.49) are showing positive correlations with vaginal pH.

### 3.4 Alpha diversity pre-vs-post treatment

**Figure 4:**
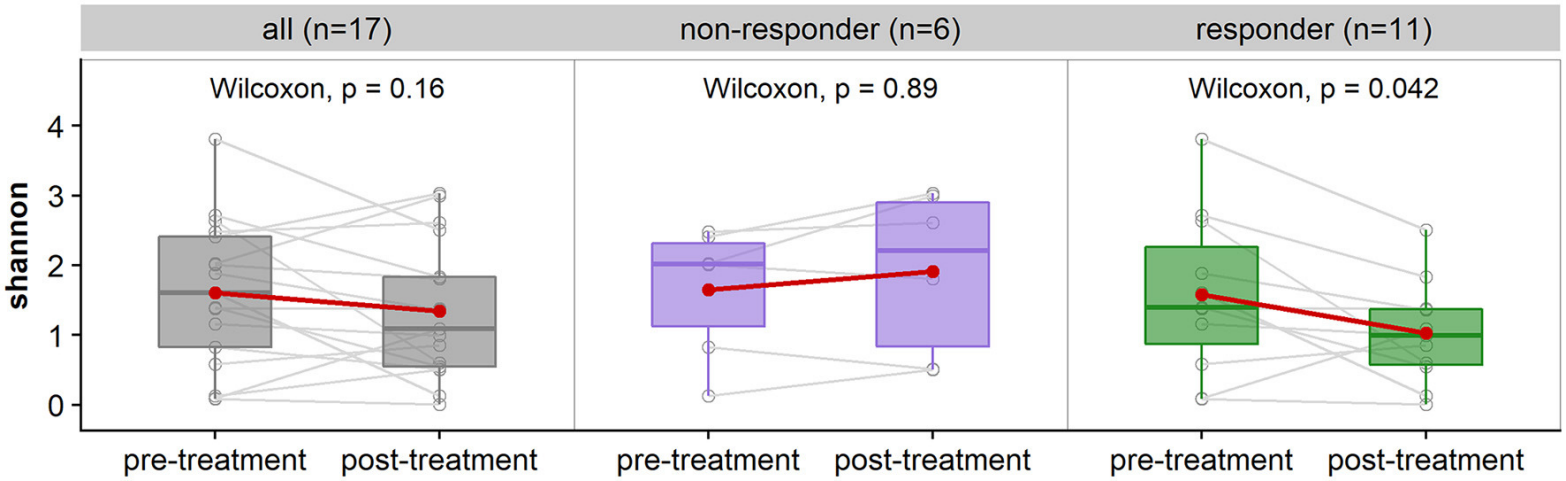
Comparison of alpha-diversity measured with Shannon index of patient's pre-treatment vs. post-treatment. **(Left)** shows the comparison for all subjects (*n* = 17 subjects, 34 samples), **(middle)** is a breakdown of non-responders (*n* = 6 subjects, 12 samples), and **(right)** are the responders (*n* = 11 subjects, 22 samples). There is no significant difference in alpha-diversity for non-responders (*p* > 0.05), and significant difference for responders with a decrease in alpha-diversity post-treatment (*p* < 0.05).

Thirty-seven of the 44 samples passed rarefaction normalization of which 34 are paired samples belonging to 17 patients. These were used in the comparison of alpha-diversity. No statistically significant difference in alpha-diversity (Shannon index) was observed from pre-treatment to post-treatment with vaginal estrogen across all 17 patients (Figure 4). However, when considering the responder type, there was a difference observed. The 11 responders (women with reduced pH in response to vaginal estrogen) showed significant decrease in alpha-diversity (*p* = 0.042) post-treatment. The non-responders (*n* = 6) displayed no significant difference in alpha-diversity (*p* = 0.89) post-treatment (Figure 4). A repeated measures ANOVA was also performed to test for any interactions between the responder type and treatment, which was not statistically significant (*p* = 0.054).

The decrease in vaginal alpha-diversity is associated with a decrease in vaginal pH as indicated by repeated measures of correlation (coefficient = 0.62). This reading reached statistical significance (*p* = 0.006).

### 3.5 Compositional differences pre-vs-post treatment

**Figure 5:**
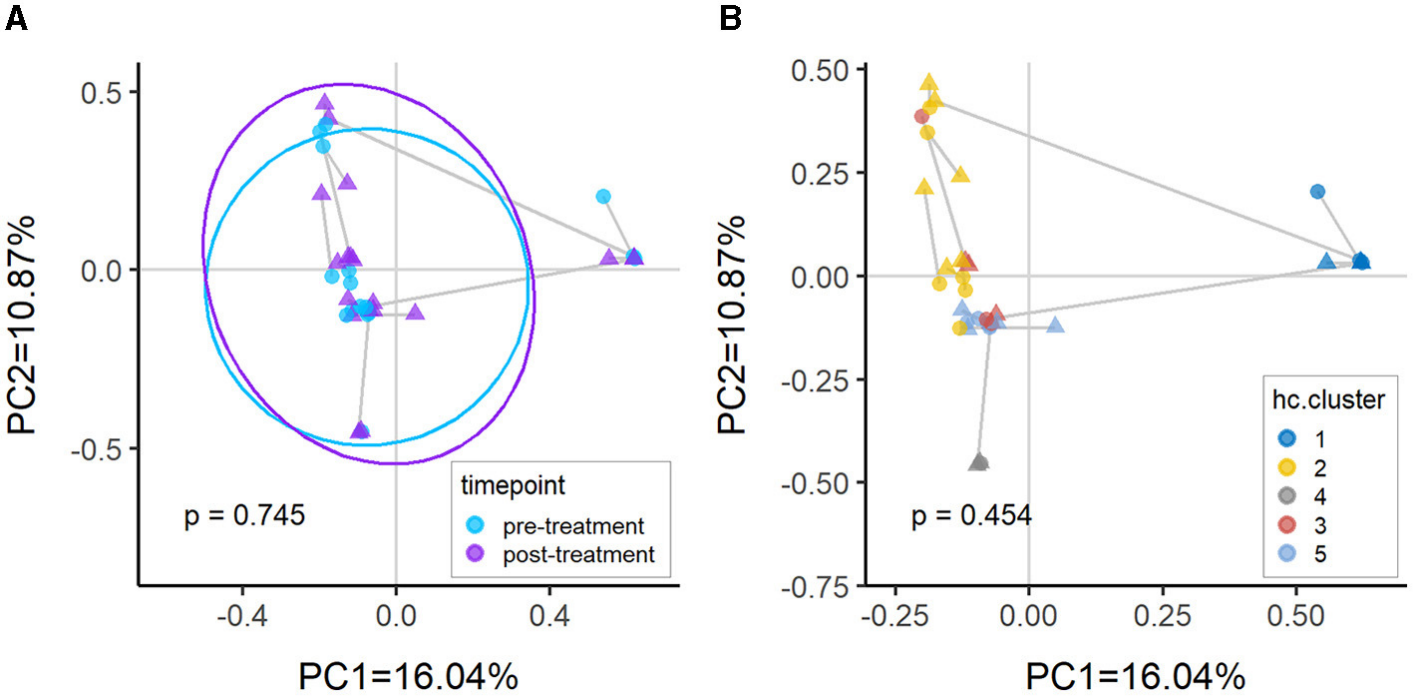
Principal coordinates analysis (PcoA) plots of Bray-Curtis dissimilarity between samples colored by **(A)** timepoint and **(B)** hierarchical clusters. There is no significant difference between pre-treatment vs. post-treatment (*p* > 0.05) as the samples are predominantly clustering by compositional similarity. The PERMANOVA *p*-values annotated in the plots are comparing pre-treatment vs. post-treatment after adjusting for **(A)** patient only and **(B)** cluster and patient.

There were no significant differences in beta-diversity measured using Bray-Curtis dissimilarity between pre- and post-treatment of vaginal estrogen. No further analyses were performed between the subdivision of responders vs. non-responders due to the small cohort size. This lack of significance is anticipated, as clusters were generally based on sample compositions (Figure 5B) rather than the treatment group (Figure 5A).

### 3.6 Responders vs. non-responders

**Figure 6:**
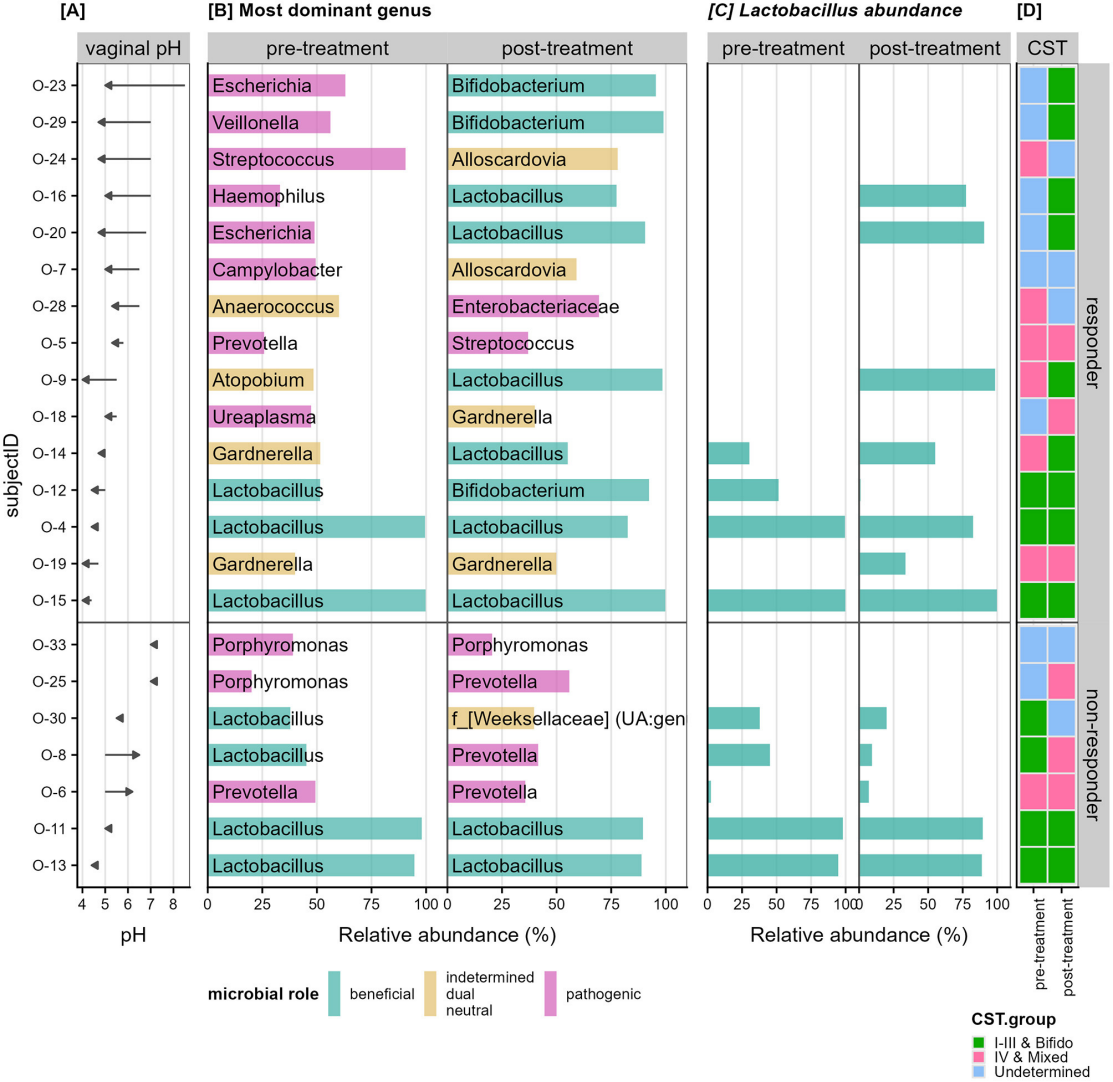
Change in vaginal pH and relative abundance of dominant taxon. **(A)** Shows the change in vaginal pH as an arrow from pre-to-post treatment per patient (row). **(B)** Shows the relative abundance of the most dominant genus for each patient separated by the pre-treatment and post-treatment samples. The bars are colored based on literature review of the roles of the dominant microbial group, whether they have been shown to be beneficial (cyan), pathogenic (pink), or indetermined (orange) in that the microbe have been shown to play either both roles depending on the composition of the community, play neither role, or not enough research has been performed. **(C)** Shows the relative abundance specific to *Lactobacillus* detected in each sample. **(D)** Shows the community state type (CST) groupings for each sample with CST I–III and Bifido colored in green; IV and mixed colored in pink and samples that do not belong to any of the known state types labeled as “undetermined” (blue).

We investigated the change in dominant taxon and Community State Type (CST) profiles according to the responder type. Figure 6 shows each patient arranged in decreasing order of vaginal pH change with the 15 responders in the top panel and seven non-responders in the bottom panel. Responders had a mean vaginal pH difference of −1.31 (SD = 0.96), while non-responders had a mean vaginal pH difference of 0.39 (SD = 0.66), showing a statistical difference (*p* = 2e−4).

Observing the dominant genus in each sample (Figure 6B) we hypothesized that women deficient in *Lactobacillus* species in their vaginal microbiota pre-treatment will show larger improvements. Of the 15 responders, 11 patients had <0.5% relative abundance *Lactobacillus* in their vaginal samples prior to treatment and were termed *Lactobacillus-*deficient (Figure 6C). The remaining four patients with more than 25% relative abundance *Lactobacillus* were termed *Lactobacillus-*present (Figure 6C). The 11 *Lactobacillus-*deficient patients had a mean vaginal pH difference of −1.65 (SD = 0.92), while the *Lactobacillus-*present patients had a mean difference of −0.40 (SD = 0.14), also showing a statistical difference (*p* = 0.004).

Independent assignment of samples based on the dominant genus per sample (Figure 6B) revealed that patients with genera associated with pathogenic roles in the pre estrogen treatment experienced larger improvement in their vaginal pH (9/15 women). In particular, the decrease in pH also correlated to a transition to beneficial taxon post treatment in five of these women. Many of these “pathogenic” type samples also align with the CST-IV or undetermined cases (Figure 6D).

However, within the responders, those that began with high levels of *Lactobacillus* species remained with high levels of beneficial dominant taxa and their vaginal pH showed minimal decrease. In contrast, the non-responders did not show any considerable change in dominant taxon post estrogen.

### 3.7 Differential abundance analysis

**Figure 7:**
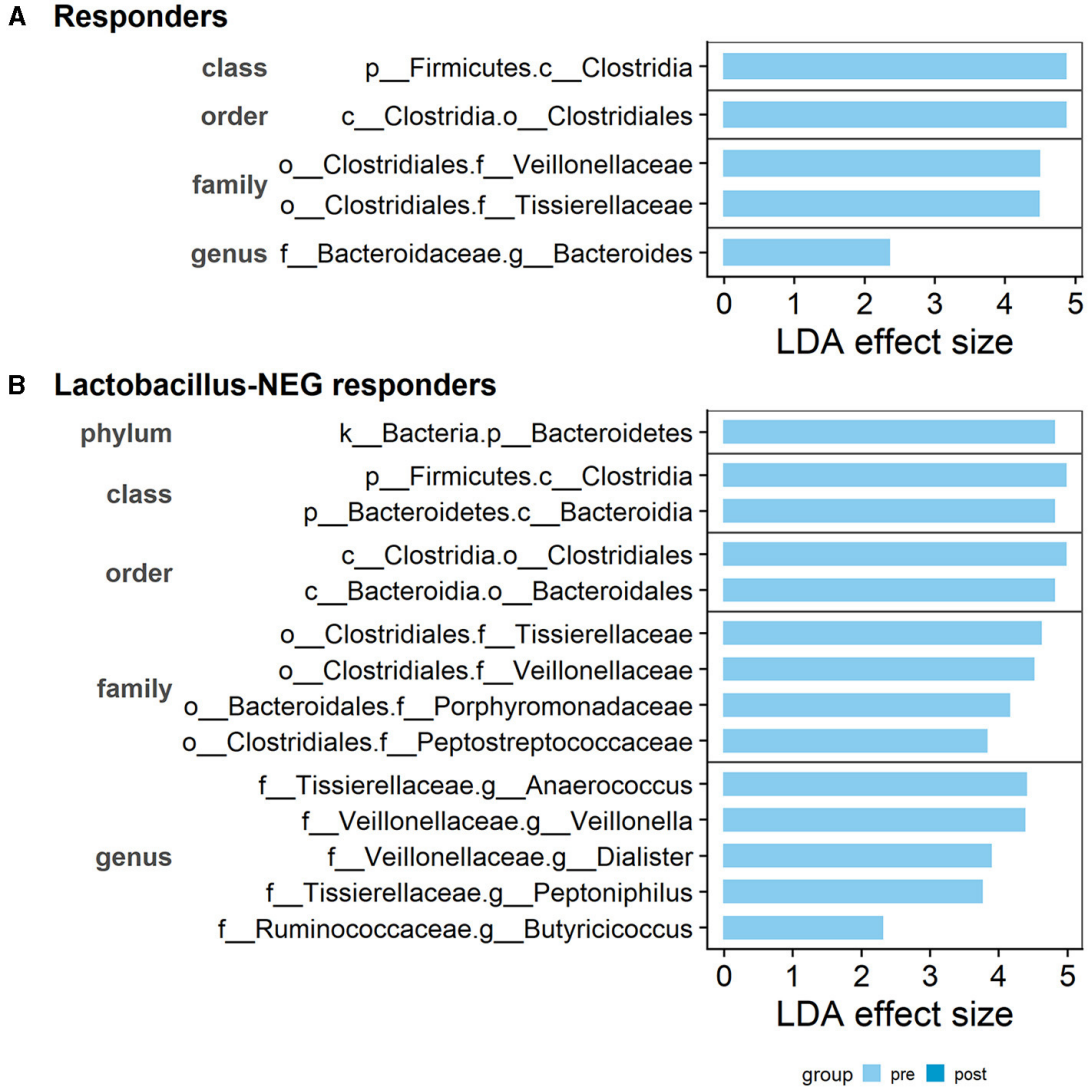
Distinct taxonomic features detected to be different between pre- and post-treatment. Within the **(A)** Responder subgroup: three distinct families, *Bacteroidaceae, Veillonellaceae*, and *Tissierellaceae* are identified as having higher relative abundance in pre-treatment than post-treatment. **(B)** Within the Lactobacillus-deficient patients, an additional three families, *Ruminococcaceae, Peptostreptococcaceae*, and *Porphyromonadaceae*, were identified as having higher relative abundance in pre-treatment than post-treatment. The LDA effect size score is provided by the LEfSe analysis. Bars are separated by the taxonomic rank and feature labels are prefixed with the rank level: p__phylum, c__class, o__order, f__family, and g__genus.

Taxonomic features which could contribute to the treatment response were investigated. These are taxa detected using the LEfSe approach as significantly different pre- vs. post-treatment. Within the 15 responders, three families, *Bacteroidaceae, Veillonellaceae*, and *Tissierellaceae* were identified as having higher relative abundance during pre-treatment (Figure 7A). There were no significant results when comparing the seven non-responders.

Following on from our hypothesis, when using the 11 *Lactobacillus*-deficient patients, we observed an increase in the list of different features. In addition to the already detected familial groups, there was another three families, *Ruminococcaceae, Peptostreptococcaceae*, and *Porphyromonadaceae*, identified as having higher relative abundance pre-treatment compared to post-treatment (Figure 7B). Similarly, there was no significant results when comparing the *Lactobacillus-*present patients. As there were no features detected as significantly different in the non-responders, the results suggests that these taxonomic features may be associated with non-protective or potentially detrimental roles.

## 4 Discussion

This novel study assesses the change in microbiota that occurs from baseline to 12 weeks post vaginal estriol use, in post-menopausal women with stress incontinence and vaginal atrophy. Here, for the first time we provide an extensive evaluation of the change in microbiota of such women after vaginal estriol treatment. In contrast to previous studies that employed the (now abandoned) systemic HRT studies (Yoshimura and Okamura, 2001; Heinemann and Reid, 2005; Shen et al., 2016), our research focused on vaginal estriol which is postulated to improve the metabolism of glycogen and restore it to pre-menopausal levels. High vaginal estrogen levels encourage the accumulation of glycogen within vaginal epithelial cells. The glycogen is subsequently metabolized by these cells (<15%) and resident bacterial population to create lactic acid, decreasing the local vaginal pH (Boskey et al., 2001). This acidic environment protects against pathogenic organisms and encourages the growth of lactic acid bacteria (LAB), which survive well in low pH environments (Witkin, 2018). Lactobacilli species are the primary producers of lactic acid. Other species such as *Bifidobacterium, Atopobium, Megasphaera, Alloscardovia, Aero-coccus, Gardnerella, Enterococcus, Streptococcus*, and *Staphylococcus* can also produce lactic acid (Zhou et al., 2004; Vitetta et al., 2017; Abedi and Hashemi, 2020).

Despite the small sample size of this study which is a limitation, our results concur with previous microbiome studies showing a significant reduction in vaginal pH with vaginal estrogen treatment (Gliniewicz et al., 2019). One of our strengths is the presence of longitudinal data from baseline to 12 weeks post-treatment. With this comparison we were able to confirm a statistically significant reduction in alpha diversity arising from vaginal estrogen treatment. The findings that women with high relative abundance of lactobacilli at baseline continued to express these results after estriol treatment (with a stable pH), suggest that these patients already have a healthy vaginal microbiota and could not improve further. Hence this allows one to understand why administering vaginal estriol is not uniformly successful in lowering the vaginal pH in all women.

In contrast, the 15 women who responded well to vaginal estrogen had largely dysbiotic community state types at baseline with dominant genus of *Prevotella, Ureaplasma, Porphyromas*, and *Streptococcus*. After vaginal estrogen, most patients showed a clear shift to either lactobacilli or other LAB bacteria such as *Bifidobacterium* (Figure 6). This finding is very interesting, as the role of non-lactobacilli species in the vagina are less well-studied. In 2004, Zhou et al. observed that when lactobacilli species were not present, other LAB increased in predominance to fill the void while still maintaining a low pH (Zhou et al., 2004). This pattern can be seen in patient O-12 where a lower vaginal pH was observed, with lactobacilli maintaining ca. 50% relative abundance (suggesting potential co-dominance between the different LAB species). In other patients such as O-23 and O-29 (Figure 6), *Bifidobacterium* replaced the role of lactobacilli while still maintaining a low pH and healthy community state type. This beneficial effect can be attributed to the D-isomer of lactic acid, which is primarily produced by lactobacilli and to lesser extent *Bifidobacterium* (Rao et al., 2018). It is observed to be more protective against vaginal dysbiosis, compared to the L-isomer counterpart produced by other LABs (Witkin et al., 2013).

While the general trend was a change from an unhealthy to a healthy community state type, not all patients in this study revealed a positive response to vaginal estrogen. In the non-responders group, patients maintained a persistently dysbiotic community state type without change in pH and had a higher level of alpha diversity with deficiencies in lactobacilli. Thus, additional mechanisms may be at play for why some women do not respond to estrogen. Risk factors such as sexual activity, BMI, hygiene practices and smoking status, which are potential factors for a dysbiotic vaginal environment may be possible (Borgdorff et al., 2017).

Furthermore, in trying to understand why some patients responded to vaginal estrogen and others did not, we examined the different taxonomic features that significantly reduced from pre to post estrogen treatment within the responder group. The most significant findings were the *Bacteroidaceae* and *Veillonellaceae* bacterial families. The *Bacteroidaceae* family consists of five genera, primarily represented by the *Bacteroides* genus which is comprised of over 40 species (Bacteroidaceae, 2012). This genus is a well-known commensal within the human gut microbiome, yet once outside of this environment, it is commonly found to be a causative agent of polymicrobial anaerobic infections, with a >19% associated mortality rate (Wexler, 2007). Bacteroides species have been observed to be associated with bacterial vaginosis (BV), a condition characterized by a loss of *Lactobacillus* spp. (Turovskiy et al., 2011; Onderdonk et al., 2016) with a concurrent substantial increase in fastidious anaerobic bacteria (Abou Chacra et al., 2021). *Bacteroides fragilis*, the most virulent species of the genus, has been identified as a causative agent of gynecological and pelvic infections, but is also involved with heart, blood, brain and gut infections (Wexler, 2007).

The *Veillonellaceae* family is comprised of 25 bacterial species across six genera (*Veillonella, Megasphaera, Dialister, Allisonella, Anaeroglobus*, and *Negativicoccus*), of which all have been found to be pathogenic in nature and involved within a large variety of human infections (Marchandin, 2014). Multiple studies have detected several *Veillonella, Megasphaera*, and *Dialister* species using molecular detection methods from vaginal swabs of women with BV, suggesting these species also play a role in vaginal dysbiosis (Fredricks et al., 2005; Srinivasan et al., 2016). A more recent study by Saliss et al. provided further evidence, observing that particular species were capable of altering the vaginal environment by increasing inflammation and cytotoxicity while simultaneously consuming lactate, and thus effectively priming the local environment for a more pathogenic residence (Salliss et al., 2021). In conjunction to these findings, our study highlights a significant reduction in abundance of these two bacterial families post-treatment, while the microbiome and vaginal pH improved. It can thus be assumed, that these bacteria are pathogenic in nature when residing within the vagina, and detrimental to the health of the vaginal microbiome. A direct competitive effect of LAB on pathogenic bacteria may be a reason for this.

After completion of recruitment for this study, during manuscript preparation, two other publications have appeared regarding the use of topical estrogen for Genitourinary Syndrome of Menopause (GUM). However, the treatments employed were quite different.

Lillemon et al. investigated the effect of a 12 week application of an estradiol-containing vaginal ring (estradiol release rate 7.5 mg/24 h) vs. placebo in 37 post-menopausal women who completed the study (*n* = 18 estradiol ring, *n* = 19 placebo ring; Lillemon et al., 2022). These authors found, on per protocol analysis, a significant increase in relative abundance of vaginal *Lactobacillus* in active treatment group, but no change in alpha diversity; they pointed out that their sample was relatively asymptomatic of GSM at baseline.

Srinivasan et al. undertook a *post-hoc* secondary analysis of women with moderate to severe GSM symptoms, but they used the “low-dose” version of estradiol (Vagifem) tablets which are placed high in the vagina (Srinivasan et al., 2022). These authors also observed a significant decrease in vaginal pH in patients on estradiol vs. placebo, with similar changes seen in the microbiota consistent with our study (among 36 women in estradiol group compared with 13 women using placebo, at a single time point). They also found that patients with high-diversity microbiota at baseline exhibited greater median change in pH compared to those with low-diversity attributes. These findings are also reiterated by a recent systematic review (Chorbinska et al., 2023).

## 5 Conclusion

In conclusion, in keeping with the recent results of Srinivasan et al. our study provides valuable insight into the mechanism whereby vaginal estrogen cream can promote growth of *Lactobacillus* and *Bifidobacterium*, yielding a more acidic vaginal pH. This should be considered a therapeutic option. However, if there is already a healthy microbiota at baseline, the estrogen treatment may not yield any further benefit and may be of lesser value.

## Data availability statement

The datasets presented in this study can be found in online repositories. The names of the repository/repositories and accession number(s) can be found at: NCBI Bioproject, accession number: PRJNA1042857.

## Ethics statement

The studies involving humans were approved by Ethics Committee the South Eastern Local District, Sydney Australia (LNR/13/POWH/685). The studies were conducted in accordance with the local legislation and institutional requirements. The participants provided their written informed consent to participate in this study.

## Author contributions

KM: Conceptualization, Funding acquisition, Methodology, Project administration, Resources, Supervision, Writing – original draft, Writing – review & editing. SO: Data curation, Formal analysis, Investigation, Methodology, Writing – original draft, Writing – review & editing. X-YC: Formal analysis, Investigation, Methodology, Project administration, Software, Validation, Visualization, Writing – original draft, Writing – review & editing. ZC: Data curation, Investigation, Methodology, Project administration, Writing – original draft, Writing – review & editing. CH: Investigation, Writing – review & editing. FE-A: Investigation, Writing – review & editing. NW: Data curation, Investigation, Writing – review & editing. EE-O: Conceptualization, Funding acquisition, Investigation, Methodology, Project administration, Resources, Supervision, Validation, Writing – review & editing.
